# How are different clusters of physical activity, sedentary, sleep, smoking, alcohol, and dietary behaviors associated with cardiometabolic health in older adults? A cross-sectional latent class analysis

**DOI:** 10.1186/s44167-023-00025-5

**Published:** 2023-08-01

**Authors:** Simone J.J.M. Verswijveren, Sara Dingle, Alan E. Donnelly, Kieran P. Dowd, Nicola D. Ridgers, Brian P. Carson, Patricia M. Kearney, Janas M. Harrington, Stephanie E. Chappel, Cormac Powell

**Affiliations:** 1 Institute of Physical Activity and Nutrition, School of Exercise and Nutrition Sciences, Deakin University, Geelong, 221 Burwood Highway, Burwood, VIC 3125 Australia; 2 Physical Activity for Health Cluster, Health Research Institute, University of Limerick, Limerick, Ireland; 3 Department of Physical Education and Sport Sciences, University of Limerick, Limerick, Ireland; 4 Health Research Institute, University of Limerick, Limerick, Ireland; 5 Department of Sport and Health Sciences, Technological University of the Shannon, Athlone Campus, Westmeath, Ireland; 6 Alliance for Research in Exercise, Nutrition and Activity (ARENA), Allied Health and Human Performance, University of South Australia, Adelaide, South Australia Australia; 7 HRB Centre for Health and Diet Research, School of Public Health, University College Cork, Cork, Ireland; 8 School of Health, Medical and Applied Sciences, Appleton Institute, Central Queensland University, Adelaide, Australia; 9 High Performance Unit, Sport Ireland, Sport Ireland Campus, Dublin, Ireland; 10 Sport and Human Performance Research Centre, Health Research Institute, University of Limerick, Limerick, Ireland

**Keywords:** Movement, Accelerometry, Sleep, Lifestyle, Clustering, Cardiometabolic health

## Abstract

**Background:**

Studies to date that investigate combined impacts of health behaviors, have rarely examined device-based movement behaviors alongside other health behaviors, such as smoking, alcohol, and sleep, on cardiometabolic health markers. The aim of this study was to identify distinct classes based on device-assessed movement behaviors (prolonged sitting, standing, stepping, and sleeping) and self-reported health behaviors (diet quality, alcohol consumption, and smoking status), and assess associations with cardiometabolic health markers in older adults.

**Methods:**

The present study is a cross-sectional secondary analysis of data from the Mitchelstown Cohort Rescreen (MCR) Study (2015–2017). In total, 1,378 older adults (aged 55–74 years) participated in the study, of whom 355 with valid activPAL3 Micro data were included in the analytical sample. Seven health behaviors (prolonged sitting, standing, stepping, sleep, diet quality, alcohol consumption, and smoking status) were included in a latent class analysis to identify groups of participants based on their distinct health behaviors. One-class through to six-class solutions were obtained and the best fit solution (i.e., optimal number of classes) was identified using a combination of best fit statistics (e.g., log likelihood, Akaike’s information criteria) and interpretability of classes. Linear regression models were used to test associations of the derived classes with cardiometabolic health markers, including body mass index, body fat, fat mass, fat-free mass, glycated hemoglobin, fasting glucose, total cholesterol, triglycerides, high-density lipoprotein cholesterol, low-density lipoprotein cholesterol, very-low-density lipoprotein cholesterol, systolic and diastolic blood pressure.

**Results:**

In total, 355 participants (89% of participants who were given the activPAL3 Micro) were included in the latent class analysis. Mean participant ages was 64.7 years and 45% were female. Two distinct classes were identified: “Healthy time-users” and “Unhealthy time-users”. These groups differed in their movement behaviors, including physical activity, prolonged sitting, and sleep. However, smoking, nutrition, and alcohol intake habits among both groups were similar. Overall, no clear associations were observed between the derived classes and cardiometabolic risk markers.

**Discussion:**

Despite having similar cardiometabolic health, two distinct clusters were identified, with differences in key behaviors such as prolonged sitting, stepping, and sleeping. This is suggestive of a complex interplay between many lifestyle behaviors, whereby one specific behavior alone cannot determine an individual’s health status. Improving the identification of the relation of multiple risk factors with health is imperative, so that effective and targeted interventions for improving health in older adults can be designed and implemented.

**Supplementary Information:**

The online version contains supplementary material available at 10.1186/s44167-023-00025-5.

## Background

Health behaviors, such as movement behaviors (including sedentary behavior [e.g., sitting] and physical activity [PA] [1]), sleep [2], diet quality [3], alcohol consumption [4], and smoking status [5], play a significant role in an individual’s health throughout life. A fundamental challenge in examining and intervening in these health behaviors is that they co-occur or cluster together [6–8]. For example, 68% of adults in England [9] and 55% in the Netherlands [10] have been reported to engage in two or more health behaviors that could be defined as being “risk behaviors” (e.g., smoking and low PA). Evidence highlights that there is a potential synergistic effect of risk behaviors, where some combinations are more damaging to health, when compared to their cumulative individual effect [9, 11]. As such, the World Health Organization (WHO) have recommended that there needs to be a focus on tackling multiple modifiable health behaviors concurrently when approaching chronic conditions prevention [12]. Despite this recommendation, public health strategies and interventions still tend to focus on health behaviors in isolation [11, 13], potentially limiting their overall effectiveness.

To design interventions that have the potential of tackling multiple health behaviors, it is imperative to know how health behaviors cluster together in groups of individuals, and if health profiles or markers differ between said groups. Whereas traditional statistical approaches to movement behaviors have tended to treat said behaviors (e.g., sedentary time, PA, etc.) as independent exposures [14], new analytical developments have allowed for the combined effects of multiple movement behaviors on health to be studied. Within the PA landscape, isotemporal substitution analysis has frequently been employed to examine the combined association of PA behaviors on a range of health markers [15–17]. More recent approaches have looked to use data-driven, person-centered approaches to better understand how a range of behaviors cluster together, and whether the identified clusters differ by health status [18–21]. The primary benefit of using these data-driven approaches is that they have the capacity to handle multidimensional and related data [16], making them a viable option to try and understand the interlinked, and complicated, relationship between the range of behaviors that individuals engage in (e.g., sleep, PA, diet, alcohol consumption) and their health status.

Within the data-drive, person-centered statistical approach to creating clusters based on individuals’ behaviors, latent class analysis (LCA) is one of the more popular methods to identify distinct clusters, and then examine how said clusters may differ, based on health outcomes [19, 21–23], while other approaches using machine-based clustering methods, such as the k-means approach [14], has also been used. The LCA approach has been used to identify clusters based on a broad range of behaviors, such as diet, PA, sitting and sleep [22], smoking status, alcohol consumption, PA, sleep, and diet [19] and diet, PA, smoking status, sleep, sitting, alcohol consumption and drug use [21]. It is clear from the existent literature, that there is a common array of behaviors included in the LCA approach (namely PA, diet, smoking status, alcohol consumption, sleep and sitting); however, the majority of research to date has tended to use self-reported measures of PA. In a recent study by Farrahi et al. [14], where a k-means approach was used to create clusters, the authors used an objective assessment of PA (accelerometry), allowing for a greater range of the PA continuum to be captured (i.e., sedentary time, light PA (LPA) and moderate-to-vigorous PA (MVPA)). Despite this improvement on the movement behaviors that were included in the clusters, said clusters did not account for other behaviors that may have an impact on an individual’s health.

To the authors’ knowledge, the majority of cluster-based studies have relied on self-reported PA, and for those studies that have used an objective assessment of PA, other potential health influencing behaviors have not been included. Therefore, the primary aim of this study was to use a LCA approach to identify clusters based on device-assessed movement behaviors (i.e., sitting, standing and stepping), alongside other potential health influencing behaviors (i.e., diet, smoking status, alcohol consumption and sleep) in older adults, and to test associations with cardiometabolic health markers.

## Methods

### Study participants

This study is a cross-sectional secondary analysis of data from the MCR Study (November 2015 - May 2017), which was a follow-up to the 2010 Cork and Kerry Diabetes and Heart Disease Study [24]. Ethical approval was obtained from the Clinical Research Ethics Committee of University College Cork (ECM 4; 07/07/2015) and the Deakin University Human Research Ethics Committee (HEAG_H 170_2019). All participants provided written informed consent, including permission to use their data for research purposes. In total, 1,378 older adults (aged 55–74 years) participated in the rescreen study. Participants attended the clinic and were asked to wear an activPAL3 Micro (PAL Technologies, Glasgow, Scotland) posture-based monitor on their right thigh for seven consecutive days. Four-hundred and forty-eight participants were offered the activPAL3 Micro. All procedures were conducted by trained research staff using standardized operating procedures, which have been described in detail previously [25]. The present manuscript is reported following the STROBE statement (Supplementary File 1 [26]).

### Measurements

#### Cardiometabolic health markers

The measurement techniques for the cardiometabolic health markers have previously been described [25]. In brief, participants provided fasted blood samples, via venipuncture, which were subsequently analyzed for glycated hemoglobin (HbA1C; mmol/mol), glucose (mmol/L), total cholesterol (mmol/L), triglycerides, high-density lipoprotein cholesterol (HDL-C; mmol/L), low-density lipoprotein cholesterol (LDL-C; mmol/L), and very-low-density lipoprotein cholesterol (VLDL-C; mmol/L) by electrochemiluminescence. Blood pressure (systolic blood pressure [SBP] and diastolic blood pressure [DBP]) was measured using an OMRON M7 Digital Blood Pressure Monitor (OMRON Healthcare, Hoofddorp, Netherlands) on the right arm, after a 5-minute rest period in a seated position. Height (cm) and body mass (kg) were measured using a portable stadiometer and an electronic scale, respectively. Participant BMI was then calculated using the standard formula (kg/m^2^). Percentage body fat (%), fat mass (kg), and fat-free mass (kg) were obtained using bioelectrical impedance (BIA).

#### Health behaviors

##### Device-assessed movement behaviors

Habitual sitting, standing, stepping, and sleep were assessed using the activPAL3 Micro, set at a 20 Hz sampling frequency. The monitor was attached to the right thigh using a nitrile sleeve and waterproof Tegaderm dressing. Participants were instructed to wear the monitor for 24 h/day, for seven consecutive days, and only remove the monitor if it were to be submerged in water. Data were downloaded using the activPAL software into event files (PAL Technologies; www.palt.com) and then processed using the ProcessingPAL software (ProcessingPAL, software v.1.3, University of Leicester, UK) with a pre-developed algorithm for adults to estimate time spent sitting, standing, stepping, and sleeping [27]. Non-wear time was excluded using previously validated activPAL wear criteria [27]. Data were only included if participants provided at least four days, including at least one weekday and one weekend day, of ≥ 10 h of waking data per day [28].

Prolonged sitting was defined as time spent in ≥ 10-minute sustained bouts with no allowance for a proportion of the bout time in higher intensities. This was based on previous work using the current dataset, which showed that time in these ≥ 10-minute bouts specifically was associated with body composition measures, lipid markers, and fasting glucose [29]. The proportion of total time spent sitting in these prolonged sitting bouts was calculated (i.e., time in bouts/total sitting time). The pre-developed algorithm identified sleep or non-wear as the longest bout per 24 h period (from noon-to-noon) that lasted at least 2 h, or as any very long bout lasting at least 5 h [27]. Sleep can register as multiple periods of sitting/lying, interspersed with real or erroneously detected posture changes, and stepping [27]; therefore, 24-hour heat-maps were visually checked and adjusted if deemed implausible by two researchers (SEC and SJJMV) (Supplementary File 2, Figure S1). Sleep was then categorized as too low (< 7 h), recommended (≥ 7–9 h) or too high (≥ 9 h) in accordance with previous literature showing that excessive sleep in older adults (i.e., ≥ 9–10 h per day) is associated with comorbidities and mortality [30].

##### Self-reported health behaviors

To assess diet quality, all participants completed a validated Food Frequency Questionnaire (FFQ), which allowed for a Dietary Approaches to Stop Hypertension (DASH) score to be computed [31]. For the FFQ, participants were asked to select their average use of food items during the previous year. The frequency of consumption of a ‘medium serving’ or a common household unit was asked for each food item and later converted into quantities using standard portion sizes. The frequency responses were ‘never or less than once a month’, ‘1–3 times/month’, ‘1 time/week’, ‘2–4 times/week’, ‘5–6 times/week’, ‘1 time/day’, ‘2–3 times/day’, ‘4–5 times/day’ or ‘≥6 times/day’ [32]. Consequently, a DASH score was derived for each participant, based on their FFQ responses. The DASH score is a composite score that is derived from standard food groups within the FFQ [33]. Briefly, for each food group, consumption was divided into quintiles and participants were classified according to their intake ranking. Consumption of healthy food components was rated on a 1–5 scale, where the higher the score, the more frequent the consumption of said food (i.e., those in quintile 1 had the lowest consumption and received a score of 1). Conversely, those in quintile 5 had the highest consumption and received a score of 5. The less-healthy dietary elements (where lower consumption is desired,) were scored on a reverse scale, with lower consumption receiving higher scores. Component scores were summed to give an overall DASH score for each participant, where a lower score indicated poorer dietary quality. For smoking status, participants were asked ‘are you a current or former smoker?’, with response options being ‘current’, ‘former’ or ‘not applicable’. For alcohol consumption behavior, participants were asked ‘how often do you have a drink containing alcohol?’, with response options being ‘monthly or less’, ‘2–4 times a month’, ‘2–3 times week’, ‘4 or more times a week’, or ‘never’. Both the smoking status and alcohol consumption behavior questions have been used with the previous cohorts of the Cork and Kerry Diabetes and Heart Disease Study, namely the Cork and Kerry cohort [34] and the Mitchelstown cohort [24]. Both sets of questions were reported using a clinical report form and a computer-assisted personal interviewing (CAPI) general health questionnaire (with a trained researcher).

#### Covariates

The clinical report form and the computer-assisted personal interviewing general health questionnaire collected age (years), sex (male/female), current employment status (employed/not employed), and reported heart conditions and medication use for blood pressure, cholesterol, and diabetes (yes/no). Being employed included being in paid part-time or full-time work, but not retirement.

### Statistical analyses

Statistical analyses were performed using Stata Version 15.0 (StataCorp, College Station, TX) and Mplus Version 8.6 [35]. Seven health behaviors (prolonged sitting, standing, stepping, sleep, diet quality, alcohol consumption, and smoking status) were included in the LCA. The LCA was originally conducted using continuous variables for prolonged sitting, standing, stepping, and diet quality, as categorizing these variables can lead to loss in richness of data. However, this resulted in issues related to model non-convergence, and thus prolonged sitting, and total volume of standing and stepping were dichotomized based on the median values for this sample (under/over median); the DASH scores were categorized into quintiles as per previous literature [36, 37]. Participants were included in the LCA if they provided valid activPAL3 Micro data (n = 355), regardless of them providing complete self-reported health behavior data (e.g., smoking, nutrition and/or alcohol intake). The missing data on the self-reported health behaviors was handled in Mplus using maximum likelihood estimation.

One-class through to six-class solutions were obtained in Mplus and the best fit solution (i.e., optimal number of classes) was identified using a combination of best fit statistics (log likelihood, Akaike’s information criteria [AIC], Bayesian information criteria [BIC], Adjusted BIC, Entropy, Lo-Mendell-Rubin (LMR) and bootstrapped likelihood ratio tests (BLRT)), class size (i.e., lowest proportion cut-off was set at 5% of the sample [38]), and interpretability of classes. Lower AIC, BIC, and adjusted BIC values indicate better model fit [39]. Entropy ranges from 0 to 1, with a value closer to 1 indicating better class separation [39]. The LMR and BLRT compare results from the solution + 1 class compared with the previous solution, and provides a p-value for determining if there is an improvement in fit for the inclusion of one more class [40]. The “best” model was identified as the model with the fewest number of classes with a better relative fit than the initial one-class model [41]. Once this model was selected, the obtained classes were used as a categorical predicting variable for use in further analyses.

Descriptive statistics (mean ± standard deviation [SD] and proportions) were obtained for the analytical sample and compared between the classes using t-tests and chi-square tests. Linear regression models were run between the classes as the categorical exposure variable and continuous cardiometabolic health markers. Only participants with complete cardiometabolic health marker and confounder data were included in these analyses (n = 222 to n = 321, depending on the outcome [63-88%]). Two differently adjusted models were used: Model 1 adjusted for age (continuous), sex and employment status (both binary); Model 2 further adjusted for reported heart conditions and medication use (all binary). Fat mass was included in Model 2 in models of non-body mass related cardiometabolic health markers (i.e., HbA1C, fasting glucose, total cholesterol, triglycerides, HDL-C, LDL-C, VLDL-C, SBP, and DBP). All assumptions for linear regression models were met. Significance was assessed at the level of p < 0.05.

## Results

### Participant characteristics

Of the 1,378 participants in the rescreen study, 399 wore the activPAL3 Micro. Forty-four participants failed to provide valid data, primarily due to too few recording days. Therefore, 355 participants (89%) were included in the LCA. Participant characteristics are presented in Table 1. These were comparable with the previously reported full sample characteristics [25].

Participants were, on average, 64.7 years old. Just under half (45%) were female and approximately half (49%) were currently in paid part- or full-time employment. Mean BMI was 28.17 kg/m^2^ and three-quarters (75%) reported having a heart condition. Half (50%) and 25% of the participants had never smoked or consumed alcohol, respectively. Total sitting time accounted for approximately 8 h per day, of which more than 6 h were in prolonged sitting bouts. Participants engaged in approximately 5 h of standing and 2 h of stepping. The median proportion of sitting time in prolonged bouts (79% of total sitting time) and the median total standing (299 min) and stepping (131 min) time, were used for dichotomization of these variables (under/over median). Almost two-thirds of participants (63%) slept between 7 and 9 h per night.

**Table 1:** Participant characteristics

	n	Mean (SD) or %
**Demographic characteristics**		
Age (years)	355	64.7 (5.3)
Sex	355	
Female	163	46%
Male	192	54%
Employment status	319	
Yes	156	49%
No	163	51%
**Cardiometabolic health markers**		
Body mass index (kg/m^2^)	348	28.17 (5.37)
Body fat (%)	348	29.54 (7.89)
Fat mass (kg)	348	23.43 (8.49)
Fat-free mass (kg)	348	55.29 (11.42)
HbA1c (mmol/mol)	351	39.99 (7.49)
Fasting glucose (mmol/L)	353	5.32 (1.29)
Total cholesterol (mmol/L)	345	5.24 (1.03)
Triglycerides (mmol/L)	345	1.23 (0.71)
HDL-C (mmol/L)	345	1.45 (0.39)
LDL-C (mmol/L)	328	3.23 (0.93)
VLDL-C (mmol/L)	284	0.55 (0.26)
Systolic blood pressure (mmHg)	354	129.03 (18.39)
Diastolic blood pressure (mmHg)	354	76.17 (10.19)
**Covariates**		
Reported heart condition	354	
Yes	266	75%
No	88	25%
Blood pressure medication	308	
Yes	111	36%
No	197	64%
Cholesterol medication	315	
Yes	132	42%
No	183	58%
Diabetes medication	286	
Yes	23	8%
No	263	92%
**Health behaviors**		
Waking activPAL3 Micro wear time (mins)	355	932.2 (65.0)
Total sitting time (mins)	355	484.6 (104.9)
Time in ≥ 10-minute sitting bouts (mins)	355	380.6 (106.9)
Proportion of total sitting time in ≥ 10-minute sitting bouts (%) ^A, C^	355	77.6 (8.9)
Total standing time (mins) ^A, C^	355	310.9 (95.5)
Total stepping time (mins) ^A, C^	355	136.7 (53.9)
Sleep (mins) ^A^	355	493.0 (67.5)
Sleep ^A^	355	
<7 h/night	50	14%
7–9 h/night	224	63%
>9 h/night	81	23%
DASH score ^A, B^	351	24.2 (5.2)
Alcohol consumption ^A^ 4 or more times a week	313 28	9%
2–3 times a week	66	21%
2–4 times a month	81	26%
Monthly or less	59	19%
Never	79	25%
Smoking status ^A^	313	
Current	28	9%
Former	128	41%
Not applicable	157	50%

HbA1c: Glycated hemoglobin; HDL-C: high-density lipoprotein cholesterol; LDL-C: low-density lipoprotein cholesterol; VLDL-C: very-low-density lipoprotein cholesterol; DASH: Dietary Approaches to Stop Hypertension.

Data were only included if participants provided at least four days of valid activity data (defined as ≥ 10 h of waking data per day) (28), and include at least one weekday and one weekend day.

^A^ These variables were categorized (if applicable) and included in the latent class analysis.

^B^ Diet was measured using the DASH diet quality score derived from the standard validated FFQ (31). The DASH scores were categorized into quintiles; a lower quintile indicates poorer diet quality.

^C^ The prolonged sitting, standing, and stepping variables were dichotomized based on the median value on these variables (under/over median).

### Latent classes of health behaviors

**Fig. 1:**
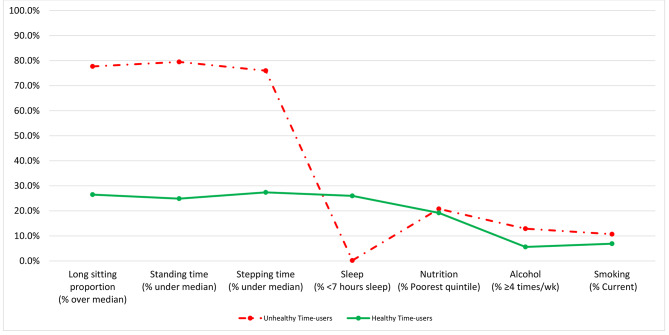
Response probabilities of categorical health behavior variables

Table 2 presents the best fit indicators for one through six class latent class solutions. The two-class solution had the lowest BIC and adjusted BIC, while the three-class solution had the lowest AIC, indicating better fits for these models compared to others. Only the two-class solution demonstrated significant LMR and BLRT results compared with the previous solution (i.e., the solution with n-1 classes; p < 0.05). Entropy was highest for the four-class solution. Taken into consideration the range of best fit statistics and the lack of meaningful additional insights from the four-class model, the two-class model was deemed the “best” and therefore the two identified classes were used to represent accumulation patterns in further analyses. Latent classes were labelled according to their distinguishing features as “Healthy time-users” and “Unhealthy time-users”. Figure 1 shows the categorical health behavior variable response probability plot representing the probability for those in each of the two classes to engage in the “unhealthiest” health behavior (i.e., over the median prolonged sitting, under the median standing and stepping, < 7 h of sleep per day, worst diet quality quintile, ≥ 4 drinks per week, and current smoker). Table 3 presents comparisons between the “Healthy time-users” and “Unhealthy time-users” for all variables. The “Healthy time-users” had significantly less total (441.6 min vs. 533.1 min) and prolonged sitting time (326.9 min vs. 441.0 min), but more total standing (362.2 min vs. 253.3 min) and stepping (160.8 min vs. 109.5 min) time, compared to the “Unhealthy time-users”. Whilst the “Unhealthy time-users” had a significantly greater amount of sleep (531.7 min vs. 458.6 min), they were also less likely to fall into the recommended 7–9 h of sleep per day category. The “Healthy time-users” had a lower proportion of individuals consuming alcohol four or more times per week (7% vs. 12%) and less current smokers (6% vs. 12%), compared to the “Unhealthy time-users”. In terms of demographics, the only observed difference was that the “Unhealthy time-users” were more likely to be currently employed compared to the “Healthy time-users” (63% vs. 40%).

**Table 2:** Best fit and diagnostic criteria for latent class models of one to six class solutions

Class	LL	AIC	BIC	Adjusted BIC	Entropy	LMR (p-value)	BLRT (p-value)	Smallest class count (n)	Smallest class size (%)
**One-class**	-2401.0	4832.1	4890.2	4842.6	N/A	N/A	N/A	355	100
**Two-class**	-2325.7	4713.5	**4833.5**	**4735.2**	0.61	**148.9 (< 0.01)**	**-2401.0 (< 0.01)**	167/188	47.0/53.0
**Three-class**	-2307.6	**4709.2**	4891.2	4742.1	0.69	35.9 (0.91)	-2325.7 (0.09)	56/199/100	15.8/56.1/28.2
**Four-class**	-2294.1	4714.2	4958.1	4758.3	**0.82**	27.1 (1.00)	-2307.8 (0.67)	89/67/143/56	25.1/18.9/40.3/15.8
**Five-class**	-2279.5	4717.0	5022.9	4772.2	0.76	29.6 (0.80)	-2294.4 (1.00)	135/74/69/41/36	38.0/20.8/19.4/11.5/10.1
**Six-class**	-2265.6	4721.3	5089.1	4787.7	0.76	40.3 (0.78)	-2286.0 (0.33)	46/81/40/30/77/81	13.0/22.8/11.3/8.4/21.7/22.8

**Bold values indicate the value corresponding to the “best” model according to each fit indicator.**

LL: Log Likelihood; AIC: Akaike’s Information Criterion; BIC: Bayesian Information Criterion; LMR: Lo-Mendell-Rubin likelihood ratio test; BLRT: Bootstrapped likelihood ratio test.

**Table 3:** Differences between health behavior related classes: “Healthy time-users” vs. “Unhealthy time-users”

	“Healthy time-users” (n = 188)		“Unhealthy time-users” (n = 167)		p-value ^A^
	n	Mean (SD) or %	n	Mean (SD) or %	
**Demographic characteristics**					
Age (years)	188	64.3 (5.3)	167	65.2 (5.4)	0.121
Sex	188		167		
Female	96	51%	97	58%	0.185
Male	92	49%	70	52%	
Employment status	169		150		
Yes	68	40%	95	63%	**< 0.001**
No	101	60%	55	37%	
**Cardiometabolic health markers**					
Body mass index (kg/m^2^)	184	27.74 (4.31)	164	28.66 (6.33)	0.112
Body fat (%)	184	28.85 (7.88)	164	30.31 (7.85)	0.086
Fat mass (kg)	184	22.38 (8.02)	164	24.60 (8.87)	**0.015**
Fat-free mass (kg)	184	54.70 (11.22)	164	55.95 (11.65)	0.311
HbA1c (mmol/mol)	186	39.34 (6.19)	165	40.73 (8.69)	0.084
Fasting glucose (mmol/L)	186	5.21 (1.32)	167	5.45 (1.25)	0.080
Total cholesterol (mmol/L)	183	5.32 (0.96)	162	5.15 (1.11)	0.132
Triglycerides (mmol/L)	183	1.14 (0.74)	162	1.32 (0.67)	**0.021**
HDL-C (mmol/L)	183	1.51 (0.39)	162	1.38 (0.38)	**0.003**
LDL-C (mmol/L)	174	3.29 (0.86)	154	3.15 (1.00)	0.170
VLDL-C (mmol/L)	153	0.53 (0.27)	131	0.57 (0.24)	0.220
Systolic blood pressure (mmHg)	188	128.88 (18.36)	166	129.21 (18.47)	0.869
Diastolic blood pressure (mmHg)	188	75.39 (9.92)	166	77.06 (10.43)	0.124
Blood pressure medication	166		142		
Yes		33%		61%	0.251
No		66%		39%	
Cholesterol medication	168		147		
Yes		65%		50%	0.194
No		35%		50%	
Diabetes medication	156		130		
Yes		5%		12%	**0.015**
No		96%		88%	
**Health behaviors**					
Waking activPAL3 Micro wear time (mins)	188	964.5 (54.2)	167	895.85 (56.6)	**< 0.001**
Total sitting time (mins)	188	441.6 (96.0)	167	533.06 (92.8)	**< 0.001**
Time in ≥ 10-minute sitting bouts (mins)	188	326.9 (92.0)	167	441.01 (88.8)	**< 0.001**
Proportion of total sitting time in ≥ 10-minute sitting bouts (%)	188	73.2 (0.1)	167	82.48 (0.1)	**< 0.001**
Proportion of total sitting time in ≥ 10-minute sitting bouts ^B^	188		167		
Under median		77%		19%	**< 0.001**
Equal or over median		23%		81%	
Total standing time (mins)	188	362.2 (81.5)	167	253.3 (75.0)	**< 0.001**
Total standing time ^B^	188		167		
Under median		21%		83%	**< 0.001**
Equal or over median		79%		17%	
Total stepping time (mins)	188	160.8 (52.6)	167	109.5 (40.9)	**< 0.001**
Total stepping time ^B^	188		167		
Under median		26%		77%	**< 0.001**
Equal or over median		74%		23%	
Sleep time (mins)	188	458.6 (56.8)	167	531.7 (56.9)	**< 0.001**
Sleep time ^B^	188		167		
<7 h/night		27%		0%	**< 0.001**
7–9 h/night		71%		54%	
>9 h/night		3%		46%	
DASH score	186	24.4 (5.1)	165	24.0 (5.2)	0.574
DASH score ^B, C^	186		165		
Quintile 1 (poorest)		19%		21%	0.815
Quintile 2		19%		21%	
Quintile 3		19%		12%	
Quintile 4		23%		18%	
Quintile 5 (best)		20%		19%	
Alcohol consumption ^B^	168		145		
4 or more times/week		7%		12%	**0.011**
2–3 times/week		17%		26%	
2–4 times/month		31%		20%	
Monthly or less		23%		14%	
Never		23%		28%	
Smoking status ^B^	168		145		
Current		6%		12%	**0.041**
Former Never		39% 55%		26% 53%	

**Bold values indicate significant differences at the level of p < 0.05.**

HbA1c: Glycated hemoglobin; HDL-C: high-density lipoprotein cholesterol; LDL-C: low-density lipoprotein cholesterol; VLDL-C: very-low-density lipoprotein cholesterol; DASH: Dietary Approaches to Stop Hypertension.

^A^ p-values were obtained through t-tests and chi-square tests for differences between classes on continuous and categorical variables, respectively.

^B^ These categorical variables were included in the latent class analysis.

^C^ Diet was measured using the DASH diet quality score derived from the standard validated FFQ (31). The DASH scores were categorized into quintiles; a lower quintile indicates poorer diet quality.

### Differences between latent classes and associations with cardiometabolic health markers

Differences in cardiometabolic health markers are described in Table 3. The “Healthy time-users” had significantly lower body fat (22.38% vs. 24.60%) and triglycerides (1.14 mmol/L vs. 1.32 mmol/L), and significantly higher HDL-C (1.51 mmol/L vs. 1.38 mmol/L), compared to the “Unhealthy time-users”. Results from these regression models (Table 4) showed that the “Healthy time-users” had lower body fat (0.08%) and higher HDL-C (0.109 mmol/L) when compared to the “Unhealthy time-users” (i.e., selected referent group). However, these associations were attenuated after adjusting for reported heart conditions, medication use, and fat mass (if applicable). No further associations between the latent classes and remaining cardiometabolic health markers were observed.

**Table 4:** Regression coefficients (β) and 95% Confidence Intervals (CIs) for associations between health behavior related classes and cardiometabolic health markers

	Model 1		Model 2	
Health behavior related classes	n	β (95% CI)	n	β (95% CI)
Body mass index (kg/m^2^)	312		251	
Unhealthy time-users		Referent		Referent
Healthy time-users		-0.051 (-1.813, 0.691)		-0.034 (-1.920, 1.120)
Body fat (%)	312		251	
Unhealthy time-users		Referent		Referent
Healthy time-users		**-0.088 (-2.718, -0.0534)**		-0.054 (-2.450, 0.700)
Fat mass (kg)	312		251	
Unhealthy time-users		Referent		Referent
Healthy time-users		-0.105 (-3.648, 0.106)		-0.061 (-3.272, 1.152)
Fat-free mass (kg)	312		251	
Unhealthy time-users		Referent		Referent
Healthy time-users		-0.023 (-1.906, 0.848)		-0.016 (-1.960, 1.244)
HbA1c (mmol/mol)	311		250	
Unhealthy time-users		Referent		Referent
Healthy time-users		-0.045 (-2.390, 1.048)		-0.008 (-1.604, 1.367)
Fasting glucose (mmol/L)	311		250	
Unhealthy time-users		Referent		Referent
Healthy time-users		-0.039 (-0.401, 0.195)		0.018 (-0.228, 0.323)
Total cholesterol (mmol/L)	304		245	
Unhealthy time-users		Referent		Referent
Healthy time-users		0.026 (-0.171, 0.278)		-0.028 (-0.278, 0.163)
Triglycerides (mmol/L)	304		245	
Unhealthy time-users		Referent		Referent
Healthy time-users		-0.069 (-0.262, 0.0581)		-0.059 (-0.227, 0.0817)
HDL-C (mmol/L)	304		245	
Unhealthy time-users		Referent		Referent
Healthy time-users		**0.109 (0.0108, 0.162)**		0.082 (-0.0198, 0.145)
LDL-C (mmol/L)	287		229	
Unhealthy time-users		Referent		Referent
Healthy time-users		0.016 (-0.186, 0.245)		-0.035 (-0.280, 0.146)
VLDL-C (mmol/L)	248		222	
Unhealthy time-users		Referent		Referent
Healthy time-users		0.001 (-0.0620, 0.0635)		-0.004 (-0.0712, 0.0667)
Systolic blood pressure (mmHg)	311		250	
Unhealthy time-users		Referent		Referent
Healthy time-users		0.041 (-2.765, 5.884)		0.026 (-4.004, 6.031)
Diastolic blood pressure (mmHg)	311		250	
Unhealthy time-users		Referent		Referent
Healthy time-users		-0.039(-3.051, 1.470)		-0.070 (-4.065, 1.141)

**Bold values indicate significant associations at the level of p < 0.05.**

HbA1c: Glycated hemoglobin; HDL-C: high-density lipoprotein cholesterol; LDL-C: low-density lipoprotein cholesterol; VLDL-C: very-low-density lipoprotein cholesterol; DASH: Dietary Approaches to Stop Hypertension

Linear regression models were conducted to analyze associations between the health behavior related classes and each of the continuous cardiometabolic health markers. Two differently adjusted models were used: Model 1 adjusted for age (continuous), sex and employment status (both binary); Model 2 further adjusted for reported heart conditions, blood pressure medication use, cholesterol medication use, and diabetes medication use (all binary). Fat mass was included in Model 2 as a covariate in models of non-body mass related cardiometabolic health markers (i.e., HbA1C, fasting glucose, total cholesterol, triglycerides, HDL-C, LDL-C, VLDL-C, SBP, and DBP).

## Discussion

The aim of this study was to identify distinct classes, based on the collection of health behaviors in a representative sample of older adults, and test associations with cardiometabolic health markers. Two classes were identified: “Healthy time-users” and “Unhealthy time-users”. When compared to the “Unhealthy time-users”, the “Healthy time-users” had lower prolonged and total sitting time, and more stepping on an average day. The “Healthy time-users” were also more likely to meet the sleep guidelines. Despite their movement behaviors being considered healthier based on previous research [6–8], “Healthy time-users” nutrition, alcohol, and smoking habits were similar compared to the “Unhealthy time-users”. No associations between the distinct classes and cardiometabolic health markers were observed in the fully adjusted models.

The findings from the current study offer interesting insights into the grouping of health behaviors associations with cardiometabolic health markers. One interesting finding is that our study only identified two distinct clusters, compared to similar studies typically reporting clustering in three or more distinct groups [14, 20, 42]. The created classes (“Healthy time-users” and “Unhealthy time-users”) suggest that movement behaviors, more distinctly than the self-reported health behaviors, such as smoking and nutrition, cluster together. This may be possible since movement behaviors, occur within a 24-hour finite period, yet other health behaviors do not have this co-dependency necessarily (i.e., it is possible to be a smoker and eat well). Comparing this directly with other data-driven clustering studies is complicated since the limited number of papers that has focused on health behaviors in older adults assessed these health behaviors differently (e.g., TV time instead of sitting time as a measure of sedentary behavior; fruit and vegetable consumption rather than an overall food score) [43]. Despite this, the current study seems to contrast the results from a self-reported study in an elderly cohort (mean age = 71 years) [44], who found that nutrition was the most distinct health behavior, and that movement behaviors were similar. On the other hand, our findings are in line with a study in ~ 40-70-year-olds [45] that found that movement behaviors (prolonged sitting and being physically inactive) clustered within classes, but alcohol intake was similar. The contrasting results between these studies, including the present work, confirms that more research is needed for identifying how different health behaviors cluster together in older adults [46]. Such information is critical to develop tailored interventions for optimizing their health.

Based on the current literature, less time spent sitting [43], more time spent standing [47] and stepping [48], and getting an appropriate amount of sleep [46], are deemed as health enabling movement behaviors. Despite these being most favorable in the “Healthy time-users”, only limited evidence was found to suggest that these were associated with cardiometabolic health markers. Since classes were comparable in terms of nutrition, alcohol, and smoking, this may suggest that, within the current cohort, these health behaviors are key for cardiometabolic health markers, regardless of their movement behaviors. This is in line with a large latent class study that included data from ≥ 500,000 middle-aged participants, that found that the clustering of poor nutrition and high alcohol intake was associated with higher odds of cardiovascular disease, compared to the clustering of physical inactivity and poor nutrition [45]. Nevertheless, the worst latent class identified for cardiovascular disease risk was the clustering of multiple health behaviors, including physical inactivity, prolonged sitting, poor nutrition, and high alcohol intake [45]. Whilst more research is needed to fully understand the complex relationship between these behaviors and health, this suggests that interventions should target multiple health behaviors simultaneously.

One important finding from the current study was that “Unhealthy time-users” were more likely to be employed compared to the “Healthy time-users”. Whilst our data does not give insight into the type of work conducted, it is possible that those employed, whether it is part-time or full-time, have less autonomy over how to spend their day in terms of sleep, PA, and prolonged sitting, compared to those not working. Though this cross-sectional study does not allow causal insights into the long-term relationship between employment and health, it does suggest that workplace interventions, rather than home interventions, may be most urgent to improve health in older adults.

A strength of the present study was the use of the posture-based activPAL3 Micro, rather than accelerometers which have typically failed to accurately classify standing from sitting [28, 49]. In addition, extensive objective health data were collected, allowing for comprehensive insights to be garnered. Nevertheless, limitations also need to be recognized. Firstly, with activPAL Micro monitors, there is the potential for wearers to change their habitual activity. Whilst previous work using this dataset [25] showed no significant difference in movement behaviors durations between week 1 and week 2, and no feedback was given to participants, this may have impacted their movement. We note that the wear time differed by approximately an hour between classes, and this is likely to influence their device-based movement behaviors. Since we did not incorporate the wear time in the latent class analysis, this may have impacted class allocation. Secondly, it is possible that the adjusted model, including additional adjustments for heart conditions, medication use, and fat mass, was too conservative and may have masked associations. Arguably, chronic conditions such as diabetes and obesity may sit along the causal pathway between the behaviors and health markers. While limited significant findings were observed, potentially due to the limited sample size (n = 355 with valid activPAL Micro data), it is important to note that we did not adjust for multiple testing. We used a classify-analyze approach, rather than a flexible model-based approach, which may have led to increased risk of bias in the classification of individuals [50]. Future studies should explore these types of data using a flexible-model-based approach. Thirdly, since more “Unhealthy time-users” compared to “Healthy time-users” were on diabetes medication, these may have normalized glucose control markers. Fourthly, the absence of an overall summary score created using several cardiometabolic health markers lacks interpretation of associations with overall cardiometabolic health. Fifthly, no direct measure of socioeconomic status was available despite this being a well-known factor for health [51, 52]. Finally, due to the cross-sectional nature of the current study, causation cannot be determined.

## Conclusions

This study identified two distinct classes with unique health behaviors: “Healthy time-users” and “Unhealthy time-users”. While these groups primarily differed in their movement behaviors (i.e., prolonged sitting, stepping, and sleep), their smoking, nutrition, and alcohol intake habits were similar. However, no associations were observed with cardiometabolic risk markers. This is suggestive of a complex interplay between many lifestyle behaviors, whereby one specific behavior alone cannot determine an individual’s health status, and therefore considering and applying a more holistic approach is required. Improving the identification of the relation of multiple risk factors with health is imperative, so that effective and targeted interventions for improving health in older adults can be designed and implemented.

## Electronic supplementary material

Below is the link to the electronic supplementary material.

Supplementary Material 1

Supplementary Material 2

Supplementary Material 3

## Data Availability

Data sharing is not applicable to this article as no datasets were generated for the current study (secondary analysis) and authors do not have permission to share these data.

## List of abbreviations

AIC: Akaike’s information criteria
BIA: Bioelectrical impedance
BIC: Bayesian information criteria
BLRT: Bootstrapped likelihood ratio test
BMI: Body mass index
CAPI: Computer-assisted personal interviewing
CIs: Confidence intervals
DASH: Dietary Approaches to Stop Hypertension
DBP: Diastolic blood pressure
FFQ: Food Frequency Questionnaire
HbA1C: Glycated hemoglobin
HDL-C: High-density lipoprotein cholesterol
LCA: Latent class analysis
LDL-C: Low-density lipoprotein cholesterol
LL: Log Likelihood
LMR: Lo-Mendell-Rubin
MCR: Mitchelstown Cohort Rescreen
MVPA: Moderate-to-vigorous physical activity
PA: Physical activity
SBP: Systolic blood pressure
SD: Standard deviation
STROBE: Strengthening The Reporting of Observational Studies in Epidemiology
VLDL-C: Very low-density lipoprotein cholesterol
WHO: World Health Organization
