# Supplementary material for: How are different clusters of physical activity, sedentary, sleep, smoking, alcohol, and dietary behaviors associated with cardiometabolic health in older adults? A cross-sectional latent class analysis
Source: J Act Sedentary Sleep Behav. 2023 Aug 1;2:16. doi: 10.1186/s44167-023-00025-5 (PMC11960331; doi:10.1186/s44167-023-00025-5)
Supplement: Supplementary file 2 — Supplementary Material 2 [file 44167_2023_25_MOESM2_ESM.docx]

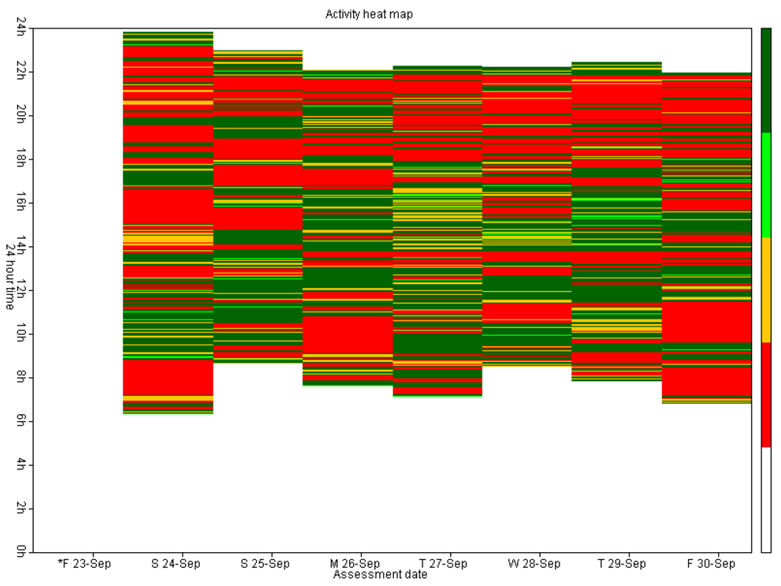
 Green = Upright stepping

Yellow = Upright standing

Red = Sitting and/or lying

Sleep

**Figure S1. Example heat-map of activity for total monitoring period**
