# Supplementary material for: How are different clusters of physical activity, sedentary, sleep, smoking, alcohol, and dietary behaviors associated with cardiometabolic health in older adults? A cross-sectional latent class analysis
Source: J Act Sedentary Sleep Behav. 2023 Aug 1;2:16. doi: 10.1186/s44167-023-00025-5 (PMC11960331; doi:10.1186/s44167-023-00025-5)
Supplement: Supplementary file 3 — Supplementary Material 3 [file 44167_2023_25_MOESM3_ESM.docx]

**Title**

How are different clusters of activity, sedentary, sleep, smoking, alcohol, and dietary behaviors associated with cardiometabolic health in older adults? A cross-sectional latent class analysis

**Authors**

Simone J.J.M. Verswijveren (PhD)^1^, Sara Dingle^1^, Alan E. Donnelly (PhD)^2,3,4^, Kieran P. Dowd (PhD)^5^, Nicola D. Ridgers (PhD)^1,6^, Brian P. Carson (PhD)^2,3,4^, Patricia M. Kearney (MD, PhD)^7^, Janas M. Harrington (PhD)^7^, Stephanie E. Chappel (PhD)^8^ and Cormac Powell (PhD)^2,9,10^

**Affiliations**

^1^ Institute of Physical Activity and Nutrition, School of Exercise and Nutrition Sciences, Deakin University, Geelong, Australia.

^2^ Physical Activity for Health Cluster, Health Research Institute, University of Limerick, Limerick, Ireland.

^3^ Department of Physical Education and Sport Sciences, University of Limerick, Limerick, Ireland.

^4^ Health Research Institute, University of Limerick, Limerick, Ireland.

^5^ Department of Sport and Health Sciences, Athlone Campus, Technological University of the Shannon, Westmeath, Ireland.

^6^ Alliance for Research in Exercise, Nutrition and Activity (ARENA), Allied Health and Human Performance, University of South Australia, Adelaide, South Australia, Australia.

^7^ HRB Centre for Health and Diet Research, School of Public Health, University College Cork, Cork, Ireland.

^8^ School of Health, Medical and Applied Sciences, Appleton Institute, Central Queensland University, Adelaide, Australia.

^9^ High Performance Unit, Sport Ireland, Sport Ireland Campus, Dublin, Ireland.

^10^ Sport and Human Performance Research Centre, Health Research Institute, University of Limerick, Limerick, Ireland.

**Corresponding author:** Dr Simone J.J.M. Verswijveren, Institute of Physical Activity and Nutrition, School of Exercise and Nutrition Sciences, Deakin University, 221 Burwood Highway, Burwood, Victoria, 3125, Australia. Tel: +61 3 9244 5224; Email: [s.verswijveren@deakin.edu.au](mailto:sjverswi@deakin.edu.au). **Abstract**

Background: Studies to date that investigate combined impacts of health behaviors, have rarely examined device-based movement behaviors alongside other health behaviors, such as smoking, alcohol, and sleep, on cardiometabolic health markers. The aim of this study was to identify distinct classes based on device-assessed movement behaviors (prolonged sitting, standing, stepping, and sleeping) and self-reported health behaviors (diet quality, alcohol consumption, and smoking status), and assess associations with cardiometabolic health markers in older adults.

Methods: The present study is a cross-sectional secondary analysis of data from the Mitchelstown Cohort Rescreen (MCR) Study (2015-2017). In total, 1,378 older adults (aged 55-74 years) participated in the study, of whom 355 with valid activPAL3 Micro data were included in the analytical sample. Seven health behaviors (prolonged sitting, standing, stepping, sleep, diet quality, alcohol consumption, and smoking status) were included in a latent class analysis to identify groups of participants based on their distinct health behaviors. One-class through to six-class solutions were obtained and the best fit solution (i.e., optimal number of classes) was identified using a combination of best fit statistics (e.g., log likelihood, Akaike’s information criteria) and interpretability of classes. Linear regression models were used to test associations of the derived classes with cardiometabolic health markers, including body mass index, body fat, fat mass, fat-free mass, glycated hemoglobin, fasting glucose, total cholesterol, triglycerides, high-density lipoprotein cholesterol, low-density lipoprotein cholesterol, very-low-density lipoprotein cholesterol, systolic and diastolic blood pressure.

Results: In total, 355 participants (89 % of participants who were given the activPAL3 Micro) were included in the latent class analysis. Mean participant ages was 64.7 years and 45 % were female. Two distinct classes were identified: “Healthy time-users” and “Unhealthy time-users”. These groups differed in their movement behaviors, including physical activity, prolonged sitting, and sleep. However, smoking, nutrition, and alcohol intake habits among both groups were similar. Overall, no clear associations were observed between the derived classes and cardiometabolic risk markers.

Discussion: Despite having similar cardiometabolic health, two distinct clusters were identified, with differences in key behaviors such as prolonged sitting, stepping, and sleeping. This is suggestive of a complex interplay between many lifestyle behaviors, whereby one specific behavior alone cannot determine an individual’s health status. Improving the identification of the relation of multiple risk factors with health is imperative, so that effective and targeted interventions for improving health in older adults can be designed and implemented.

**Key words**

Movement, accelerometry, sleep, lifestyle, clustering, cardiometabolic health

**Background**

Health behaviors, such as movement behaviors (including sedentary behavior [e.g., sitting] and physical activity [PA] (1)), sleep (2), diet quality (3), alcohol consumption (4), and smoking status (5), play a significant role in an individual’s health throughout life. A fundamental challenge in examining and intervening in these health behaviors is that they co-occur or cluster together (6-8). For example, 68 % of adults in England (9) and 55 % in the Netherlands (10) have been reported to engage in two or more health behaviors that could be defined as being “risk behaviors” (e.g., smoking and low PA). Evidence highlights that there is a potential synergistic effect of risk behaviors, where some combinations are more damaging to health, when compared to their cumulative individual effect (9, 11). As such, the World Health Organization (WHO) have recommended that there needs to be a focus on tackling multiple modifiable health behaviors concurrently when approaching chronic conditions prevention (12). Despite this recommendation, public health strategies and interventions still tend to focus on health behaviors in isolation (11, 13), potentially limiting their overall effectiveness.

To design interventions that have the potential of tackling multiple health behaviors, it is imperative to know how health behaviors cluster together in groups of individuals, and if health profiles or markers differ between said groups. Whereas traditional statistical approaches to movement behaviors have tended to treat said behaviors (e.g., sedentary time, PA, etc.) as independent exposures (14), new analytical developments have allowed for the combined effects of multiple movement behaviors on health to be studied. Within the PA landscape, isotemporal substitution analysis has frequently been employed to examine the combined association of PA behaviors on a range of health markers (15-17). More recent approaches have looked to use data-driven, person-centered approaches to better understand how a range of behaviors cluster together, and whether the identified clusters differ by health status (18-21). The primary benefit of using these data-driven approaches is that they have the capacity to handle multidimensional and related data (16), making them a viable option to try and understand the interlinked, and complicated, relationship between the range of behaviors that individuals engage in (e.g., sleep, PA, diet, alcohol consumption) and their health status.

Within the data-drive, person-centered statistical approach to creating clusters based on individuals’ behaviors, latent class analysis (LCA) is one of the more popular methods to identify distinct clusters, and then examine how said clusters may differ, based on health outcomes (19, 21-23), while other approaches using machine-based clustering methods, such as the k-means approach (14), has also been used. The LCA approach has been used to identify clusters based on a broad range of behaviors, such as diet, PA, sitting and sleep (22), smoking status, alcohol consumption, PA, sleep, and diet (19) and diet, PA, smoking status, sleep, sitting, alcohol consumption and drug use (21). It is clear from the existent literature, that there is a common array of behaviors included in the LCA approach (namely PA, diet, smoking status, alcohol consumption, sleep and sitting); however, the majority of research to date has tended to use self-reported measures of PA. In a recent study by Farrahi et al. (14), where a k-means approach was used to create clusters, the authors used an objective assessment of PA (accelerometry), allowing for a greater range of the PA continuum to be captured (i.e., sedentary time, light PA (LPA) and moderate-to-vigorous PA (MVPA)). Despite this improvement on the movement behaviors that were included in the clusters, said clusters did not account for other behaviors that may have an impact on an individual’s health.

To the authors’ knowledge, the majority of cluster-based studies have relied on self-reported PA, and for those studies that have used an objective assessment of PA, other potential health influencing behaviors have not been included. Therefore, the primary aim of this study was to use a LCA approach to identify clusters based on device-assessed movement behaviors (i.e., sitting, standing and stepping), alongside other potential health influencing behaviors (i.e., diet, smoking status, alcohol consumption and sleep) in older adults, and to test associations with cardiometabolic health markers.

**Methods**

***Study participants***

This study is a cross-sectional secondary analysis of data from the MCR Study (November 2015 - May 2017), which was a follow-up to the 2010 Cork and Kerry Diabetes and Heart Disease Study (24). Ethical approval was obtained from the Clinical Research Ethics Committee of University College Cork (ECM 4; 07/07/2015) and the Deakin University Human Research Ethics Committee (HEAG_H 170_2019). All participants provided written informed consent, including permission to use their data for research purposes. In total, 1,378 older adults (aged 55-74 years) participated in the rescreen study. Participants attended the clinic and were asked to wear an activPAL3 Micro (PAL Technologies, Glasgow, Scotland) posture-based monitor on their right thigh for seven consecutive days. Four-hundred and forty-eight participants were offered the activPAL3 Micro. All procedures were conducted by trained research staff using standardized operating procedures, which have been described in detail previously (25). The present manuscript is reported following the STROBE statement (**Supplementary File 1** (26)).

***Measurements***

Cardiometabolic health markers

The measurement techniques for the cardiometabolic health markers have previously been described (25). In brief, participants provided fasted blood samples, via venipuncture, which were subsequently analyzed for glycated hemoglobin (HbA1C; mmol/mol), glucose (mmol/L), total cholesterol (mmol/L), triglycerides, high-density lipoprotein cholesterol (HDL-C; mmol/L), low-density lipoprotein cholesterol (LDL-C; mmol/L), and very-low-density lipoprotein cholesterol (VLDL-C; mmol/L) by electrochemiluminescence. Blood pressure (systolic blood pressure [SBP] and diastolic blood pressure [DBP]) was measured using an OMRON M7 Digital Blood Pressure Monitor (OMRON Healthcare, Hoofddorp, Netherlands) on the right arm, after a 5-minute rest period in a seated position. Height (cm) and body mass (kg) were measured using a portable stadiometer and an electronic scale, respectively. Participant BMI was then calculated using the standard formula (kg/m^2^). Percentage body fat (%), fat mass (kg), and fat-free mass (kg) were obtained using bioelectrical impedance (BIA).

Health behaviors

*Device-assessed movement behaviors*

Habitual sitting, standing, stepping, and sleep were assessed using the activPAL3 Micro, set at a 20Hz sampling frequency. The monitor was attached to the right thigh using a nitrile sleeve and waterproof Tegaderm dressing. Participants were instructed to wear the monitor for 24 hours/day, for seven consecutive days, and only remove the monitor if it were to be submerged in water. Data were downloaded using the activPAL software into event files (PAL Technologies; [www.palt.com](file:///C:\Users\cpowell\Downloads\www.palt.com)) and then processed using the ProcessingPAL software (ProcessingPAL, software v.1.3, University of Leicester, UK) with a pre-developed algorithm for adults to estimate time spent sitting, standing, stepping, and sleeping (27). Non-wear time was excluded using previously validated activPAL wear criteria (27). Data were only included if participants provided at least four days, including at least one weekday and one weekend day, of ≥10h of waking data per day (28).

Prolonged sitting was defined as time spent in ≥10-minute sustained bouts with no allowance for a proportion of the bout time in higher intensities. This was based on previous work using the current dataset, which showed that time in these ≥10-minute bouts specifically was associated with body composition measures, lipid markers, and fasting glucose (29). The proportion of total time spent sitting in these prolonged sitting bouts was calculated (i.e., time in bouts/total sitting time). The pre-developed algorithm identified sleep or non-wear as the longest bout per 24h period (from noon-to-noon) that lasted at least 2 hours, or as any very long bout lasting at least 5 hours (27). Sleep can register as multiple periods of sitting/lying, interspersed with real or erroneously detected posture changes, and stepping (27); therefore, 24-hour heat-maps were visually checked and adjusted if deemed implausible by two researchers (SEC and SJJMV) (**Supplementary File 2, Figure S1**). Sleep was then categorized as too low (<7 hours), recommended (≥7-9 hours) or too high (≥9 hours) in accordance with previous literature showing that excessive sleep in older adults (i.e., ≥9–10 h per day) is associated with comorbidities and mortality (30).

*Self-reported health behaviors*

To assess diet quality, all participants completed a validated Food Frequency Questionnaire (FFQ), which allowed for a Dietary Approaches to Stop Hypertension (DASH) score to be computed (31). For the FFQ, participants were asked to select their average use of food items during the previous year. The frequency of consumption of a ‘medium serving’ or a common household unit was asked for each food item and later converted into quantities using standard portion sizes. The frequency responses were ‘never or less than once a month’, ‘1–3 times/month’, ‘1 time/week’, ‘2–4 times/week’, ‘5–6 times/week’, ‘1 time/day’, ‘2–3 times/day’, ‘4–5 times/day’ or ‘≥6 times/day’ (32). Consequently, a DASH score was derived for each participant, based on their FFQ responses. The DASH score is a composite score that is derived from standard food groups within the FFQ (33). Briefly, for each food group, consumption was divided into quintiles and participants were classified according to their intake ranking. Consumption of healthy food components was rated on a 1-5 scale, where the higher the score, the more frequent the consumption of said food (i.e., those in quintile 1 had the lowest consumption and received a score of 1). Conversely, those in quintile 5 had the highest consumption and received a score of 5. The less-healthy dietary elements (where lower consumption is desired,) were scored on a reverse scale, with lower consumption receiving higher scores. Component scores were summed to give an overall DASH score for each participant, where a lower score indicated poorer dietary quality. For smoking status, participants were asked ‘are you a current or former smoker?’, with response options being ‘current’, ‘former’ or ‘not applicable’. For alcohol consumption behavior, participants were asked ‘how often do you have a drink containing alcohol?’, with response options being ‘monthly or less’, ‘2-4 times a month’, ‘2-3 times week’, ‘4 or more times a week’, or ‘never’. Both the smoking status and alcohol consumption behavior questions have been used with the previous cohorts of the Cork and Kerry Diabetes and Heart Disease Study, namely the Cork and Kerry cohort (34) and the Mitchelstown cohort (24). Both sets of questions were reported using a clinical report form and a computer-assisted personal interviewing (CAPI) general health questionnaire (with a trained researcher).

Covariates

The clinical report form and the computer-assisted personal interviewing general health questionnaire collected age (years), sex (male/female), current employment status (employed/not employed), and reported heart conditions and medication use for blood pressure, cholesterol, and diabetes (yes/no). Being employed included being in paid part-time or full-time work, but not retirement.

***Statistical analyses***

Statistical analyses were performed using Stata Version 15.0 (StataCorp, College Station, TX) and Mplus Version 8.6 (35). Seven health behaviors (prolonged sitting, standing, stepping, sleep, diet quality, alcohol consumption, and smoking status) were included in the LCA. The LCA was originally conducted using continuous variables for prolonged sitting, standing, stepping, and diet quality, as categorizing these variables can lead to loss in richness of data. However, this resulted in issues related to model non-convergence, and thus prolonged sitting, and total volume of standing and stepping were dichotomized based on the median values for this sample (under/over median); the DASH scores were categorized into quintiles as per previous literature (36, 37). Participants were included in the LCA if they provided valid activPAL3 Micro data (n= 355), regardless of them providing complete self-reported health behavior data (e.g., smoking, nutrition and/or alcohol intake). The missing data on the self-reported health behaviors was handled in Mplus using maximum likelihood estimation.

One-class through to six-class solutions were obtained in Mplus and the best fit solution (i.e., optimal number of classes) was identified using a combination of best fit statistics (log likelihood, Akaike’s information criteria [AIC], Bayesian information criteria [BIC], Adjusted BIC, Entropy, Lo-Mendell-Rubin (LMR) and bootstrapped likelihood ratio tests (BLRT)), class size (i.e., lowest proportion cut-off was set at 5 % of the sample (38)), and interpretability of classes. Lower AIC, BIC, and adjusted BIC values indicate better model fit (39). Entropy ranges from 0-1, with a value closer to 1 indicating better class separation (39). The LMR and BLRT compare results from the solution +1 class compared with the previous solution, and provides a p-value for determining if there is an improvement in fit for the inclusion of one more class (40). The “best” model was identified as the model with the fewest number of classes with a better relative fit than the initial one-class model (41). Once this model was selected, the obtained classes were used as a categorical predicting variable for use in further analyses.

Descriptive statistics (mean ± standard deviation [SD] and proportions) were obtained for the analytical sample and compared between the classes using t-tests and chi-square tests. Linear regression models were run between the classes as the categorical exposure variable and continuous cardiometabolic health markers. Only participants with complete cardiometabolic health marker and confounder data were included in these analyses (n=222 to n=321, depending on the outcome [63 %-88 %]). Two differently adjusted models were used: Model 1 adjusted for age (continuous), sex and employment status (both binary); Model 2 further adjusted for reported heart conditions and medication use (all binary). Fat mass was included in Model 2 in models of non-body mass related cardiometabolic health markers (i.e., HbA1C, fasting glucose, total cholesterol, triglycerides, HDL-C, LDL-C, VLDL-C, SBP, and DBP). All assumptions for linear regression models were met. Significance was assessed at the level of p<0.05.

**Results**

***Participant characteristics***

Of the 1,378 participants in the rescreen study, 399 wore the activPAL3 Micro. Forty-four participants failed to provide valid data, primarily due to too few recording days. Therefore, 355 participants (89 %) were included in the LCA. Participant characteristics are presented in **Table 1**. These were comparable with the previously reported full sample characteristics (25).

Participants were, on average, 64.7 years old. Just under half (45 %) were female and approximately half (49 %) were currently in paid part- or full-time employment. Mean BMI was 28.17 kg/m^2^ and three-quarters (75 %) reported having a heart condition. Half (50 %) and 25 % of the participants had never smoked or consumed alcohol, respectively. Total sitting time accounted for approximately 8 hours per day, of which more than 6 hours were in prolonged sitting bouts. Participants engaged in approximately 5 hours of standing and 2 hours of stepping. The median proportion of sitting time in prolonged bouts (79 % of total sitting time) and the median total standing (299 min) and stepping (131 min) time, were used for dichotomization of these variables (under/over median). Almost two-thirds of participants (63 %) slept between 7-9 h per night.

| **Table 1. Participant characteristics** | | |
| --- | --- | --- |
|  | **n** | **Mean (SD) or %** |
| **Demographic characteristics** | | |
| Age (years) | 355 | 64.7 (5.3) |
| Sex  Female  Male | 355  163  192 | 46 %  54 % |
| Employment status  Yes  No | 319  156  163 | 49 %  51 % |
| **Cardiometabolic health markers** | | |
| Body mass index (kg/m^2^) | 348 | 28.17 (5.37) |
| Body fat (%) | 348 | 29.54 (7.89) |
| Fat mass (kg) | 348 | 23.43 (8.49) |
| Fat-free mass (kg) | 348 | 55.29 (11.42) |
| HbA1c (mmol/mol) | 351 | 39.99 (7.49) |
| Fasting glucose (mmol/L) | 353 | 5.32 (1.29) |
| Total cholesterol (mmol/L) | 345 | 5.24 (1.03) |
| Triglycerides (mmol/L) | 345 | 1.23 (0.71) |
| HDL-C (mmol/L) | 345 | 1.45 (0.39) |
| LDL-C (mmol/L) | 328 | 3.23 (0.93) |
| VLDL-C (mmol/L) | 284 | 0.55 (0.26) |
| Systolic blood pressure (mmHg) | 354 | 129.03 (18.39) |
| Diastolic blood pressure (mmHg) | 354 | 76.17 (10.19) |
| **Covariates** | | |
| Reported heart condition  Yes  No | 354  266  88 | 75 %  25 % |
| Blood pressure medication  Yes  No | 308  111  197 | 36 %  64 % |
| Cholesterol medication  Yes  No | 315  132  183 | 42 %  58 % |
| Diabetes medication  Yes  No | 286  23  263 | 8 %  92 % |
| **Health behaviors** | | |
| Waking activPAL3 Micro wear time (mins) | 355 | 932.2 (65.0) |
| Total sitting time (mins) | 355 | 484.6 (104.9) |
| Time in ≥10-minute sitting bouts (mins) | 355 | 380.6 (106.9) |
| Proportion of total sitting time in ≥10-minute sitting bouts (%) ^A, C^ | 355 | 77.6 (8.9) |
| Total standing time (mins) ^A, C^ | 355 | 310.9 (95.5) |
| Total stepping time (mins) ^A, C^ | 355 | 136.7 (53.9) |
| Sleep (mins) ^A^ | 355 | 493.0 (67.5) |
| Sleep ^A^  <7 h/night  7-9 h/night  >9 h/night | 355  50  224  81 | 14 %  63 %  23 % |
| DASH score ^A, B^ | 351 | 24.2 (5.2) |
| Alcohol consumption ^A^  4 or more times a week  2-3 times a week  2-4 times a month  Monthly or less  Never | 313  28  66  81  59  79 | 9 %  21 %  26 %  19 %  25 % |
| Smoking status ^A^  Current  Former  Not applicable | 313  28  128  157 | 9 %  41 %  50 % |
| HbA1c: Glycated hemoglobin; HDL-C: high-density lipoprotein cholesterol; LDL-C: low-density lipoprotein cholesterol; VLDL-C: very-low-density lipoprotein cholesterol; DASH: Dietary Approaches to Stop Hypertension.  Data were only included if participants provided at least four days of valid activity data (defined as ≥10h of waking data per day) (28), and include at least one weekday and one weekend day.  ^A^ These variables were categorized (if applicable) and included in the latent class analysis.  ^B^ Diet was measured using the DASH diet quality score derived from the standard validated FFQ (31). The DASH scores were categorized into quintiles; a lower quintile indicates poorer diet quality.  ^C^ The prolonged sitting, standing, and stepping variables were dichotomized based on the median value on these variables (under/over median). | | |

***Latent classes of health behaviors***

**Table 2** presents the best fit indicators for one through six class latent class solutions. The two-class solution had the lowest BIC and adjusted BIC, while the three-class solution had the lowest AIC, indicating better fits for these models compared to others. Only the two-class solution demonstrated significant LMR and BLRT results compared with the previous solution (i.e., the solution with n-1 classes; p<0.05). Entropy was highest for the four-class solution. Taken into consideration the range of best fit statistics and the lack of meaningful additional insights from the four-class model, the two-class model was deemed the “best” and therefore the two identified classes were used to represent accumulation patterns in further analyses.

Latent classes were labelled according to their distinguishing features as “Healthy time-users” and “Unhealthy time-users”. **Figure 1** shows the categorical health behavior variable response probability plot representing the probability for those in each of the two classes to engage in the “unhealthiest” health behavior (i.e., over the median prolonged sitting, under the median standing and stepping, <7 hours of sleep per day, worst diet quality quintile, ≥4 drinks per week, and current smoker). **Table 3** presents comparisons between the “Healthy time-users” and “Unhealthy time-users” for all variables. The “Healthy time-users” had significantly less total (441.6 mins vs. 533.1 mins) and prolonged sitting time (326.9 mins vs. 441.0 mins), but more total standing (362.2 mins vs. 253.3 mins) and stepping (160.8 mins vs. 109.5 mins) time, compared to the “Unhealthy time-users”. Whilst the “Unhealthy time-users” had a significantly greater amount of sleep (531.7 mins vs. 458.6 mins), they were also less likely to fall into the recommended 7-9 hours of sleep per day category. The “Healthy time-users” had a lower proportion of individuals consuming alcohol four or more times per week (7 % vs. 12 %) and less current smokers (6 % vs. 12 %), compared to the “Unhealthy time-users”. In terms of demographics, the only observed difference was that the “Unhealthy time-users” were more likely to be currently employed compared to the “Healthy time-users” (63 % vs. 40 %).

| **Table 2. Best fit and diagnostic criteria for latent class models of one to six class solutions** | | | | | | | | | |
| --- | --- | --- | --- | --- | --- | --- | --- | --- | --- |
| **Class** | **LL** | **AIC** | **BIC** | **Adjusted BIC** | **Entropy** | **LMR (p-value)** | **BLRT (p-value)** | **Smallest class count (n)** | **Smallest class size (%)** |
| **One-class** | -2401.0 | 4832.1 | 4890.2 | 4842.6 | N/A | N/A | N/A | 355 | 100 |
| **Two-class** | -2325.7 | 4713.5 | **4833.5** | **4735.2** | 0.61 | **148.9 (<0.01)** | **-2401.0 (<0.01)** | 167/188 | 47.0/53.0 |
| **Three-class** | -2307.6 | **4709.2** | 4891.2 | 4742.1 | 0.69 | 35.9 (0.91) | -2325.7 (0.09) | 56/199/100 | 15.8/56.1/28.2 |
| **Four-class** | -2294.1 | 4714.2 | 4958.1 | 4758.3 | **0.82** | 27.1 (1.00) | -2307.8 (0.67) | 89/67/143/56 | 25.1/18.9/40.3/15.8 |
| **Five-class** | -2279.5 | 4717.0 | 5022.9 | 4772.2 | 0.76 | 29.6 (0.80) | -2294.4 (1.00) | 135/74/69/41/36 | 38.0/20.8/19.4/11.5/10.1 |
| **Six-class** | -2265.6 | 4721.3 | 5089.1 | 4787.7 | 0.76 | 40.3 (0.78) | -2286.0 (0.33) | 46/81/40/30/77/81 | 13.0/22.8/11.3/8.4/21.7/22.8 |
| **Bold values indicate the value corresponding to the “best” model according to each fit indicator.**  LL: Log Likelihood; AIC: Akaike’s Information Criterion; BIC: Bayesian Information Criterion; LMR: Lo-Mendell-Rubin likelihood ratio test; BLRT: Bootstrapped likelihood ratio test. | | | | | | | | | |

**Figure 1. Response probabilities of categorical health behavior variables.**

| **Table 3. Differences between health behavior related classes: “Healthy time-users” vs. “Unhealthy time-users”** | | | | | |
| --- | --- | --- | --- | --- | --- |
|  | **“Healthy time-users” (n=188)** | | **“Unhealthy time-users” (n=167)** | | **p-value ^A^** |
|  | **n** | **Mean (SD) or %** | **n** | **Mean (SD) or %** |  |
| **Demographic characteristics** | | | | | |
| Age (years) | 188 | 64.3 (5.3) | 167 | 65.2 (5.4) | 0.121 |
| Sex  Female  Male | 188  96  92 | 51 %  49 % | 167  97  70 | 58 %  52 % | 0.185 |
| Employment status  Yes  No | 169  68  101 | 40 %  60 % | 150  95  55 | 63 %  37 % | **<0.001** |
| **Cardiometabolic health markers** | | | | | |
| Body mass index (kg/m^2^) | 184 | 27.74 (4.31) | 164 | 28.66 (6.33) | 0.112 |
| Body fat (%) | 184 | 28.85 (7.88) | 164 | 30.31 (7.85) | 0.086 |
| Fat mass (kg) | 184 | 22.38 (8.02) | 164 | 24.60 (8.87) | **0.015** |
| Fat-free mass (kg) | 184 | 54.70 (11.22) | 164 | 55.95 (11.65) | 0.311 |
| HbA1c (mmol/mol) | 186 | 39.34 (6.19) | 165 | 40.73 (8.69) | 0.084 |
| Fasting glucose (mmol/L) | 186 | 5.21 (1.32) | 167 | 5.45 (1.25) | 0.080 |
| Total cholesterol (mmol/L) | 183 | 5.32 (0.96) | 162 | 5.15 (1.11) | 0.132 |
| Triglycerides (mmol/L) | 183 | 1.14 (0.74) | 162 | 1.32 (0.67) | **0.021** |
| HDL-C (mmol/L) | 183 | 1.51 (0.39) | 162 | 1.38 (0.38) | **0.003** |
| LDL-C (mmol/L) | 174 | 3.29 (0.86) | 154 | 3.15 (1.00) | 0.170 |
| VLDL-C (mmol/L) | 153 | 0.53 (0.27) | 131 | 0.57 (0.24) | 0.220 |
| Systolic blood pressure (mmHg) | 188 | 128.88 (18.36) | 166 | 129.21 (18.47) | 0.869 |
| Diastolic blood pressure (mmHg) | 188 | 75.39 (9.92) | 166 | 77.06 (10.43) | 0.124 |
| Blood pressure medication  Yes  No | 166 | 33 %  66 % | 142 | 61 %  39 % | 0.251 |
| Cholesterol medication  Yes  No | 168 | 65 %  35 % | 147 | 50 %  50 % | 0.194 |
| Diabetes medication  Yes  No | 156 | 5 %  96 % | 130 | 12 %  88 % | **0.015** |
| **Health behaviors** | | | | | |
| Waking activPAL3 Micro wear time (mins) | 188 | 964.5 (54.2) | 167 | 895.85 (56.6) | **<0.001** |
| Total sitting time (mins) | 188 | 441.6 (96.0) | 167 | 533.06 (92.8) | **<0.001** |
| Time in ≥10-minute sitting bouts (mins) | 188 | 326.9 (92.0) | 167 | 441.01 (88.8) | **<0.001** |
| Proportion of total sitting time in ≥10-minute sitting bouts (%) | 188 | 73.2 (0.1) | 167 | 82.48 (0.1) | **<0.001** |
| Proportion of total sitting time in ≥10-minute sitting bouts ^B^  Under median  Equal or over median | 188 | 77 %  23 % | 167 | 19 %  81 % | **<0.001** |
| Total standing time (mins) | 188 | 362.2 (81.5) | 167 | 253.3 (75.0) | **<0.001** |
| Total standing time ^B^  Under median  Equal or over median | 188 | 21 %  79 % | 167 | 83 %  17 % | **<0.001** |
| Total stepping time (mins) | 188 | 160.8 (52.6) | 167 | 109.5 (40.9) | **<0.001** |
| Total stepping time ^B^  Under median  Equal or over median | 188 | 26 %  74 % | 167 | 77 %  23 % | **<0.001** |
| Sleep time (mins) | 188 | 458.6 (56.8) | 167 | 531.7 (56.9) | **<0.001** |
| Sleep time ^B^  <7 h/night  7-9 h/night  >9 h/night | 188 | 27 %  71 %  3 % | 167 | 0 %  54 %  46 % | **<0.001** |
| DASH score | 186 | 24.4 (5.1) | 165 | 24.0 (5.2) | 0.574 |
| DASH score ^B, C^  Quintile 1 (poorest)  Quintile 2  Quintile 3  Quintile 4  Quintile 5 (best) | 186 | 19 %  19 %  19 %  23 %  20 % | 165 | 21 %  21 %  12 %  18 %  19 % | 0.815 |
| Alcohol consumption ^B^  4 or more times/week  2-3 times/week  2-4 times/month  Monthly or less  Never | 168 | 7 %  17 %  31 %  23 %  23 % | 145 | 12 %  26 %  20 %  14 %  28 % | **0.011** |
| Smoking status ^B^  Current  Former  Never | 168 | 6 %  39 %  55 % | 145 | 12 %  26 %  53 % | **0.041** |
| **Bold values indicate significant differences at the level of p<0.05.**  HbA1c: Glycated hemoglobin; HDL-C: high-density lipoprotein cholesterol; LDL-C: low-density lipoprotein cholesterol; VLDL-C: very-low-density lipoprotein cholesterol; DASH: Dietary Approaches to Stop Hypertension.  ^A^ p-values were obtained through t-tests and chi-square tests for differences between classes on continuous and categorical variables, respectively.  ^B^ These categorical variables were included in the latent class analysis.  ^C^ Diet was measured using the DASH diet quality score derived from the standard validated FFQ (31). The DASH scores were categorized into quintiles; a lower quintile indicates poorer diet quality. | | | | | |

***Differences between latent classes and associations with cardiometabolic health markers***

Differences in cardiometabolic health markers are described in **Table 3**. The “Healthy time-users” had significantly lower body fat (22.38 % vs. 24.60 %) and triglycerides (1.14 mmol/L vs. 1.32 mmol/L), and significantly higher HDL-C (1.51 mmol/L vs. 1.38 mmol/L), compared to the “Unhealthy time-users”. Results from these regression models (**Table 4**) showed that the “Healthy time-users” had lower body fat (0.08 %) and higher HDL-C (0.109 mmol/L) when compared to the “Unhealthy time-users" (i.e., selected referent group). However, these associations were attenuated after adjusting for reported heart conditions, medication use, and fat mass (if applicable). No further associations between the latent classes and remaining cardiometabolic health markers were observed.

| **Table 4. Regression coefficients (β) and 95 % Confidence Intervals (CIs) for associations between health behavior related classes and cardiometabolic health markers** | | | | |
| --- | --- | --- | --- | --- |
|  | **Model 1** | | **Model 2** | |
| **Health behavior related classes** | **n** | **β (95 % CI)** | **n** | **β (95 % CI)** |
| Body mass index (kg/m^2^)  Unhealthy time-users  Healthy time-users | 312 | Referent  -0.051 (-1.813, 0.691) | 251 | Referent  -0.034 (-1.920, 1.120) |
| Body fat (%)  Unhealthy time-users  Healthy time-users | 312 | Referent  **-0.088 (-2.718, -0.0534)** | 251 | Referent  -0.054 (-2.450, 0.700) |
| Fat mass (kg)  Unhealthy time-users  Healthy time-users | 312 | Referent  -0.105 (-3.648, 0.106) | 251 | Referent  -0.061 (-3.272, 1.152) |
| Fat-free mass (kg)  Unhealthy time-users  Healthy time-users | 312 | Referent  -0.023 (-1.906, 0.848) | 251 | Referent  -0.016 (-1.960, 1.244) |
| HbA1c (mmol/mol)  Unhealthy time-users  Healthy time-users | 311 | Referent  -0.045 (-2.390, 1.048) | 250 | Referent  -0.008 (-1.604, 1.367) |
| Fasting glucose (mmol/L)  Unhealthy time-users  Healthy time-users | 311 | Referent  -0.039 (-0.401, 0.195) | 250 | Referent  0.018 (-0.228, 0.323) |
| Total cholesterol (mmol/L)  Unhealthy time-users  Healthy time-users | 304 | Referent  0.026 (-0.171, 0.278) | 245 | Referent  -0.028 (-0.278, 0.163) |
| Triglycerides (mmol/L)  Unhealthy time-users  Healthy time-users | 304 | Referent  -0.069 (-0.262, 0.0581) | 245 | Referent  -0.059 (-0.227, 0.0817) |
| HDL-C (mmol/L)  Unhealthy time-users  Healthy time-users | 304 | Referent  **0.109 (0.0108, 0.162)** | 245 | Referent  0.082 (-0.0198, 0.145) |
| LDL-C (mmol/L)  Unhealthy time-users  Healthy time-users | 287 | Referent  0.016 (-0.186, 0.245) | 229 | Referent  -0.035 (-0.280, 0.146) |
| VLDL-C (mmol/L)  Unhealthy time-users  Healthy time-users | 248 | Referent  0.001 (-0.0620, 0.0635) | 222 | Referent  -0.004 (-0.0712, 0.0667) |
| Systolic blood pressure (mmHg)  Unhealthy time-users  Healthy time-users | 311 | Referent  0.041 (-2.765, 5.884) | 250 | Referent  0.026 (-4.004, 6.031) |
| Diastolic blood pressure (mmHg)  Unhealthy time-users  Healthy time-users | 311 | Referent  -0.039(-3.051, 1.470) | 250 | Referent  -0.070 (-4.065, 1.141) |
| **Bold values indicate significant associations at the level of p<0.05.**  HbA1c: Glycated hemoglobin; HDL-C: high-density lipoprotein cholesterol; LDL-C: low-density lipoprotein cholesterol; VLDL-C: very-low-density lipoprotein cholesterol; DASH: Dietary Approaches to Stop Hypertension  Linear regression models were conducted to analyze associations between the health behavior related classes and each of the continuous cardiometabolic health markers. Two differently adjusted models were used: Model 1 adjusted for age (continuous), sex and employment status (both binary); Model 2 further adjusted for reported heart conditions, blood pressure medication use, cholesterol medication use, and diabetes medication use (all binary). Fat mass was included in Model 2 as a covariate in models of non-body mass related cardiometabolic health markers (i.e., HbA1C, fasting glucose, total cholesterol, triglycerides, HDL-C, LDL-C, VLDL-C, SBP, and DBP). | | | | |

**Discussion**

The aim of this study was to identify distinct classes, based on the collection of health behaviors in a representative sample of older adults, and test associations with cardiometabolic health markers. Two classes were identified: “Healthy time-users” and “Unhealthy time-users”. When compared to the “Unhealthy time-users", the “Healthy time-users" had lower prolonged and total sitting time, and more stepping on an average day. The “Healthy time-users” were also more likely to meet the sleep guidelines. Despite their movement behaviors being considered healthier based on previous research (6-8), “Healthy time-users” nutrition, alcohol, and smoking habits were similar compared to the “Unhealthy time-users”. No associations between the distinct classes and cardiometabolic health markers were observed in the fully adjusted models.

The findings from the current study offer interesting insights into the grouping of health behaviors associations with cardiometabolic health markers. One interesting finding is that our study only identified two distinct clusters, compared to similar studies typically reporting clustering in three or more distinct groups (14, 20, 42). The created classes (“Healthy time-users” and “Unhealthy time-users”) suggest that movement behaviors, more distinctly than the self-reported health behaviors, such as smoking and nutrition, cluster together. This may be possible since movement behaviors, occur within a 24-hour finite period, yet other health behaviors do not have this co-dependency necessarily (i.e., it is possible to be a smoker and eat well). Comparing this directly with other data-driven clustering studies is complicated since the limited number of papers that has focused on health behaviors in older adults assessed these health behaviors differently (e.g., TV time instead of sitting time as a measure of sedentary behavior; fruit and vegetable consumption rather than an overall food score) (43). Despite this, the current study seems to contrast the results from a self-reported study in an elderly cohort (mean age = 71 years) (44), who found that nutrition was the most distinct health behavior, and that movement behaviors were similar. On the other hand, our findings are in line with a study in ~40-70-year-olds (45) that found that movement behaviors (prolonged sitting and being physically inactive) clustered within classes, but alcohol intake was similar. The contrasting results between these studies, including the present work, confirms that more research is needed for identifying how different health behaviors cluster together in older adults (46). Such information is critical to develop tailored interventions for optimizing their health.

Based on the current literature, less time spent sitting (43), more time spent standing (47) and stepping (48), and getting an appropriate amount of sleep (46), are deemed as health enabling movement behaviors. Despite these being most favorable in the “Healthy time-users", only limited evidence was found to suggest that these were associated with cardiometabolic health markers. Since classes were comparable in terms of nutrition, alcohol, and smoking, this may suggest that, within the current cohort, these health behaviors are key for cardiometabolic health markers, regardless of their movement behaviors. This is in line with a large latent class study that included data from ≥500,000 middle-aged participants, that found that the clustering of poor nutrition and high alcohol intake was associated with higher odds of cardiovascular disease, compared to the clustering of physical inactivity and poor nutrition (45). Nevertheless, the worst latent class identified for cardiovascular disease risk was the clustering of multiple health behaviors, including physical inactivity, prolonged sitting, poor nutrition, and high alcohol intake (45). Whilst more research is needed to fully understand the complex relationship between these behaviors and health, this suggests that interventions should target multiple health behaviors simultaneously.

One important finding from the current study was that “Unhealthy time-users” were more likely to be employed compared to the “Healthy time-users”. Whilst our data does not give insight into the type of work conducted, it is possible that those employed, whether it is part-time or full-time, have less autonomy over how to spend their day in terms of sleep, PA, and prolonged sitting, compared to those not working. Though this cross-sectional study does not allow causal insights into the long-term relationship between employment and health, it does suggest that workplace interventions, rather than home interventions, may be most urgent to improve health in older adults.

A strength of the present study was the use of the posture-based activPAL3 Micro, rather than accelerometers which have typically failed to accurately classify standing from sitting (28, 49). In addition, extensive objective health data were collected, allowing for comprehensive insights to be garnered. Nevertheless, limitations also need to be recognized. Firstly, with activPAL Micro monitors, there is the potential for wearers to change their habitual activity. Whilst previous work using this dataset (25) showed no significant difference in movement behaviors durations between week 1 and week 2, and no feedback was given to participants, this may have impacted their movement. We note that the wear time differed by approximately an hour between classes, and this is likely to influence their device-based movement behaviors. Since we did not incorporate the wear time in the latent class analysis, this may have impacted class allocation. Secondly, it is possible that the adjusted model, including additional adjustments for heart conditions, medication use, and fat mass, was too conservative and may have masked associations. Arguably, chronic conditions such as diabetes and obesity may sit along the causal pathway between the behaviors and health markers. While limited significant findings were observed, potentially due to the limited sample size (n=355 with valid activPAL Micro data), it is important to note that we did not adjust for multiple testing. We used a classify-analyze approach, rather than a flexible model-based approach, which may have led to increased risk of bias in the classification of individuals (50). Future studies should explore these types of data using a flexible-model-based approach. Thirdly, since more “Unhealthy time-users” compared to “Healthy time-users” were on diabetes medication, these may have normalized glucose control markers. Fourthly, the absence of an overall summary score created using several cardiometabolic health markers lacks interpretation of associations with overall cardiometabolic health. Fifthly, no direct measure of socioeconomic status was available despite this being a well-known factor for health (51, 52). Finally, due to the cross-sectional nature of the current study, causation cannot be determined.

**Conclusions**

This study identified two distinct classes with unique health behaviors: “Healthy time-users” and “Unhealthy time-users”. While these groups primarily differed in their movement behaviors (i.e., prolonged sitting, stepping, and sleep), their smoking, nutrition, and alcohol intake habits were similar. However, no associations were observed with cardiometabolic risk markers. This is suggestive of a complex interplay between many lifestyle behaviors, whereby one specific behavior alone cannot determine an individual’s health status, and therefore considering and applying a more holistic approach is required. Improving the identification of the relation of multiple risk factors with health is imperative, so that effective and targeted interventions for improving health in older adults can be designed and implemented.

**List of abbreviations**

AIC - Akaike’s information criteria

BIA - Bioelectrical impedance

BIC - Bayesian information criteria

BLRT - Bootstrapped likelihood ratio test

BMI – Body mass index

CAPI - Computer-assisted personal interviewing

CIs - Confidence intervals

DASH - Dietary Approaches to Stop Hypertension

DBP – Diastolic blood pressure

FFQ - Food Frequency Questionnaire

HbA1C – Glycated hemoglobin

HDL-C – High-density lipoprotein cholesterol

LCA – Latent class analysis

LDL-C - Low-density lipoprotein cholesterol

LL - Log Likelihood

LMR - Lo-Mendell-Rubin

MCR – Mitchelstown Cohort Rescreen

MVPA – Moderate-to-vigorous physical activity

PA – Physical activity

SBP – Systolic blood pressure

SD – Standard deviation

STROBE - Strengthening The Reporting of Observational Studies in Epidemiology

VLDL-C – Very low-density lipoprotein cholesterol

WHO – World Health Organization

**Declarations**

***Ethics approval and consent to participate***

Ethical approval was obtained from the Clinical Research Ethics Committee of University College Cork (ECM 4; 07/07/2015) and the Deakin University Human Research Ethics Committee (HEAG_H 170_2019). All participants provided written informed consent, including permission to use their data for research purposes.

***Consent for publication***

Not applicable

***Availability of data and materials***

Data sharing is not applicable to this article as no datasets were generated for the current study (secondary analysis) and authors do not have permission to share these data.

***Competing interests***

The authors declare that they have no competing interests.

***Funding***

SJJMV is supported by an Alfred Deakin Postdoctoral Research Fellowship. NDR was supported by a National Heart Foundation of Australia Future Leader Fellowship (ID 101895). CP, AED, BPC, and KPD were supported through the University of Limerick Department of Physical Education and Sport Sciences Postgraduate Scholarship Programme (2013-2017). PMK and JMH were funded through the Health Research Board Centre for Health and Diet Research (HRB 2007/2013). SD is supported by a Deakin University Postgraduate Research Scholarship.

***Authors' contributions***

AED, BPC, PMK, JMH and CP were involved in the design of the MCR Study. AED, KPD, BPC, PMK, JMH and CP were involved in the original data collection. SJJMV, SD, AED, NDR and CP conceived the study idea. SJJMV, SD, SEC and CP conducted the analyses. SJJMV, SD, AED, KPD, NDR, BPC, SEC and CP interpreted the results. All authors were involved in manuscript preparation and editing. All authors read and approved the final version of the manuscript, prior to submission.

***Acknowledgements***

The authors would like to thank the LivingHealth Clinic staff and the Mitchelstown Research Team for their involvement in the data collection, as well as the MCR Study participants. The authors would also like to specifically thank Professor Ivan J. Perry (University College Cork) for their work in setting up the MCR Study.

**References**

1. Pratt M, Ramirez Varela A, Salvo D, Kohl Iii HW, Ding D. Attacking the pandemic of physical inactivity: what is holding us back? British journal of sports medicine. 2020;54(13):760-2.

2. Fan M, Sun D, Zhou T, Heianza Y, Lv J, Li L, et al. Sleep patterns, genetic susceptibility, and incident cardiovascular disease: a prospective study of 385 292 UK biobank participants. European heart journal. 2020;41(11):1182-9.

3. Micha R, Peñalvo JL, Cudhea F, Imamura F, Rehm CD, Mozaffarian D. Association Between Dietary Factors and Mortality From Heart Disease, Stroke, and Type 2 Diabetes in the United States. JAMA : the journal of the American Medical Association. 2017;317(9):912-24.

4. Burton R, Sheron N. No level of alcohol consumption improves health. Lancet. 2018;392(10152):987-8.

5. Lee PN. The effect of reducing the number of cigarettes smoked on risk of lung cancer, COPD, cardiovascular disease and FEV(1)--a review. Regulatory toxicology and pharmacology : RTP. 2013;67(3):372-81.

6. Meader N, King K, Moe-Byrne T, Wright K, Graham H, Petticrew M, et al. A systematic review on the clustering and co-occurrence of multiple risk behaviours. BMC public health. 2016;16:657.

7. Coups EJ, Gaba A, Orleans CT. Physician screening for multiple behavioral health risk factors. American journal of preventive medicine. 2004;27(2 Suppl):34-41.

8. McAloney K, Graham H, Law C, Platt L. A scoping review of statistical approaches to the analysis of multiple health-related behaviours. Preventive medicine. 2013;56(6):365-71.

9. Poortinga W. The prevalence and clustering of four major lifestyle risk factors in an English adult population. Preventive medicine. 2007;44(2):124-8.

10. Schuit AJ, van Loon AJ, Tijhuis M, Ocké M. Clustering of lifestyle risk factors in a general adult population. Preventive medicine. 2002;35(3):219-24.

11. Conry MC, Morgan K, Curry P, McGee H, Harrington J, Ward M, et al. The clustering of health behaviours in Ireland and their relationship with mental health, self-rated health and quality of life. BMC public health. 2011;11:692.

12. 2013 World Health Organisation. Cardiovascular diseases (CVDs). Fact sheet no. 317. World Health Organisation.

13. Noble N, Paul C, Turon H, Oldmeadow C. Which modifiable health risk behaviours are related? A systematic review of the clustering of Smoking, Nutrition, Alcohol and Physical activity ('SNAP') health risk factors. Preventive medicine. 2015;81:16-41.

14. Farrahi V, Rostami M, Dumuid D, Chastin SFM, Niemelä M, Korpelainen R, et al. Joint Profiles of Sedentary Time and Physical Activity in Adults and Their Associations with Cardiometabolic Health. Med Sci Sports Exerc. 2022;54(12):2118-28.

15. Grgic J, Dumuid D, Bengoechea EG, Shrestha N, Bauman A, Olds T, et al. Health outcomes associated with reallocations of time between sleep, sedentary behaviour, and physical activity: a systematic scoping review of isotemporal substitution studies. Int J Behav Nutr Phys Act. 2018;15(1):69.

16. Migueles JH, Aadland E, Andersen LB, Brønd JC, Chastin SF, Hansen BH, et al. GRANADA consensus on analytical approaches to assess associations with accelerometer-determined physical behaviours (physical activity, sedentary behaviour and sleep) in epidemiological studies. British journal of sports medicine. 2022;56(7):376-84.

17. Dumuid D, Pedišić Ž, Stanford TE, Martín-Fernández JA, Hron K, Maher CA, et al. The compositional isotemporal substitution model: A method for estimating changes in a health outcome for reallocation of time between sleep, physical activity and sedentary behaviour. Statistical methods in medical research. 2019;28(3):846-57.

18. Gupta N, Hallman DM, Dumuid D, Vij A, Rasmussen CL, Jørgensen MB, et al. Movement behavior profiles and obesity: a latent profile analysis of 24-h time-use composition among Danish workers. International journal of obesity. 2020;44(2):409-17.

19. Kukreti S, Yu T, Chiu PW, Strong C. Clustering of Modifiable Behavioral Risk Factors and Their Association with All-Cause Mortality in Taiwan's Adult Population: a Latent Class Analysis. International journal of behavioral medicine. 2022;29(5):565-74.

20. del Pozo Cruz B, McGregor DE, del Pozo Cruz J, Buman MP, Palarea-Albaladejo J, Alfonso-Rosa RM, et al. Integrating Sleep, Physical Activity, and Diet Quality to Estimate All-Cause Mortality Risk: A Combined Compositional Clustering and Survival Analysis of the National Health and Nutrition Examination Survey 2005–2006 Cycle. American Journal of Epidemiology. 2020;189(10):1057-64.

21. Hutchesson MJ, Duncan MJ, Oftedal S, Ashton LM, Oldmeadow C, Kay-Lambkin F, et al. Latent Class Analysis of Multiple Health Risk Behaviors among Australian University Students and Associations with Psychological Distress. Nutrients. 2021;13(2).

22. Oftedal S, Vandelanotte C, Duncan MJ. Patterns of Diet, Physical Activity, Sitting and Sleep Are Associated with Socio-Demographic, Behavioural, and Health-Risk Indicators in Adults. International journal of environmental research and public health. 2019;16(13).

23. Oftedal S, Kolt GS, Holliday EG, Stamatakis E, Vandelanotte C, Brown WJ, et al. Associations of health-behavior patterns, mental health and self-rated health. Preventive medicine. 2019;118:295-303.

24. Kearney PM, Harrington JM, Mc Carthy VJ, Fitzgerald AP, Perry IJ. Cohort profile: The Cork and Kerry Diabetes and Heart Disease Study. International journal of epidemiology. 2013;42(5):1253-62.

25. Powell C, Browne LD, Carson BP, Dowd KP, Perry IJ, Kearney PM, et al. Use of Compositional Data Analysis to Show Estimated Changes in Cardiometabolic Health by Reallocating Time to Light-Intensity Physical Activity in Older Adults. Sports medicine (Auckland, NZ). 2020;50(1):205-17.

26. von Elm E, Altman DG, Egger M, Pocock SJ, Gøtzsche PC, Vandenbroucke JP. The Strengthening the Reporting of Observational Studies in Epidemiology (STROBE) statement: guidelines for reporting observational studies. Journal of clinical epidemiology. 2008;61(4):344-9.

27. Winkler EA, Bodicoat DH, Healy GN, Bakrania K, Yates T, Owen N, et al. Identifying adults' valid waking wear time by automated estimation in activPAL data collected with a 24 h wear protocol. Physiological measurement. 2016;37(10):1653-68.

28. Edwardson CL, Winkler EAH, Bodicoat DH, Yates T, Davies MJ, Dunstan DW, et al. Considerations when using the activPAL monitor in field-based research with adult populations. Journal of Sport and Health Science. 2016.

29. Verswijveren SJJM, Powell C, Chappel SE, Ridgers ND, Carson BP, Dowd KP, et al. The Influence of Sitting, Standing, and Stepping Bouts on Cardiometabolic Health Markers in Older Adults. Journal of Aging and Physical Activity. 2022;30(1):114-22.

30. Ross R, Chaput J-P, Giangregorio LM, Janssen I, Saunders TJ, Kho ME, et al. Canadian 24-Hour Movement Guidelines for Adults aged 18–64 years and Adults aged 65 years or older: an integration of physical activity, sedentary behaviour, and sleep. Applied Physiology, Nutrition, and Metabolism. 2020;45(10 (Suppl. 2)):S57-S102.

31. Harrington J, Fitzgerald AP, Layte R, Lutomski J, Molcho M, Perry IJ. Sociodemographic, health and lifestyle predictors of poor diets. Public health nutrition. 2011;14(12):2166-75.

32. Harrington J, Perry I, Lutomski J, Morgan K, McGee H, Shelley E, et al. 2007: Survey of Lifestyle, Attitudes and Nutrition in Ireland. Dietary Habits of the Irish Population. Dublin: Department of Health and Children; 2008.

33. Fung TT, Chiuve SE, McCullough ML, Rexrode KM, Logroscino G, Hu FB. Adherence to a DASH-style diet and risk of coronary heart disease and stroke in women. Archives of internal medicine. 2008;168(7):713-20.

34. Creagh D, Neilson S, Collins A, Colwell N, Hinchion R, Drew C, et al. Established cardiovascular disease and CVD risk factors in a primary care population of middle-aged Irish men and women. Irish medical journal. 2002;95(10):298-301.

35. Muthén LK, Muthén B. Mplus user's guide: statistical analysis with latent variables. Los Angeles, CA: Muthén & Muthén; 2017.

36. Soltani S, Arablou T, Jayedi A, Salehi-Abargouei A. Adherence to the dietary approaches to stop hypertension (DASH) diet in relation to all-cause and cause-specific mortality: a systematic review and dose-response meta-analysis of prospective cohort studies. Nutrition journal. 2020;19(1):37.

37. Parker KE, Salmon J, Brown HL, Villanueva K, Timperio A. Typologies of adolescent activity related health behaviours. Journal of Science and Medicine in Sport. 2019;22(3):319-23.

38. Nasserinejad K, van Rosmalen J, de Kort W, Lesaffre E. Comparison of Criteria for Choosing the Number of Classes in Bayesian Finite Mixture Models. PLoS One. 2017;12(1):e0168838.

39. Weller BE, Bowen NK, Faubert SJ. Latent Class Analysis: A Guide to Best Practice. Journal of Black Psychology. 2020;46(4):287-311.

40. Nylund KL, Asparouhov T, Muthén BO. Deciding on the Number of Classes in Latent Class Analysis and Growth Mixture Modeling: A Monte Carlo Simulation Study. Structural Equation Modeling: A Multidisciplinary Journal. 2007;14(4):535-69.

41. Masyn KE. Latent Class Analysis and Finite Mixture Modeling. In: Little TD, editor. The Oxford Handbook of Quantitative Methods in Psychology: Vol 2: Statistical Analysis: Oxford University Press; 2013.

42. de Vries H, van 't Riet J, Spigt M, Metsemakers J, van den Akker M, Vermunt JK, et al. Clusters of lifestyle behaviors: results from the Dutch SMILE study. Preventive medicine. 2008;46(3):203-8.

43. Cabanas-Sánchez V, Guallar-Castillón P, Higueras-Fresnillo S, Rodríguez-Artalejo F, Martínez-Gómez D. Changes in Sitting Time and Cardiovascular Mortality in Older Adults. American journal of preventive medicine. 2018;54(3):419-22.

44. Thuany M, Vieira D, Santos AS, Malchrowicz-Mosko E, Gomes TN. Perspectives on Movement and Eating Behaviours in Brazilian Elderly: An Analysis of Clusters Associated with Disease Outcomes. Aging Dis. 2022;13(5):1413-20.

45. Tegegne TK, Islam SMS, Maddison R. Effects of lifestyle risk behaviour clustering on cardiovascular disease among UK adults: latent class analysis with distal outcomes. Sci Rep. 2022;12(1):17349.

46. Kakinami L, O'Loughlin EK, Brunet J, Dugas EN, Constantin E, Sabiston CM, et al. Associations between physical activity and sedentary behavior with sleep quality and quantity in young adults. Sleep health. 2017;3(1):56-61.

47. van der Ploeg HP, Chey T, Ding D, Chau JY, Stamatakis E, Bauman AE. Standing time and all-cause mortality in a large cohort of Australian adults. Preventive medicine. 2014;69:187-91.

48. Adams B, Fidler K, Demoes N, Aguiar EJ, Ducharme SW, McCullough AK, et al. Cardiometabolic thresholds for peak 30-min cadence and steps/day. PLoS One. 2019;14(8):e0219933.

49. Kozey-Keadle S, Libertine A, Lyden K, Staudenmayer J, Freedson PS. Validation of Wearable Monitors for Assessing Sedentary Behavior. Medicine and Science in Sports and Exercise. 2011;43(8):1561-7.

50. Lanza ST, Tan X, Bray BC. Latent Class Analysis With Distal Outcomes: A Flexible Model-Based Approach. Struct Equ Modeling. 2013;20(1):1-26.

51. Dalstra JA, Kunst AE, Borrell C, Breeze E, Cambois E, Costa G, et al. Socioeconomic differences in the prevalence of common chronic diseases: an overview of eight European countries. International journal of epidemiology. 2005;34(2):316-26.

52. Stringhini S, Carmeli C, Jokela M, Avendaño M, McCrory C, d'Errico A, et al. Socioeconomic status, non-communicable disease risk factors, and walking speed in older adults: multi-cohort population based study. BMJ (Clinical research ed). 2018;360:k1046.
